# Social determinants of health priorities of state Medicaid programs

**DOI:** 10.1186/s12913-019-3977-5

**Published:** 2019-03-14

**Authors:** Deena J. Chisolm, Daniel L. Brook, Mary S. Applegate, Kelly J. Kelleher

**Affiliations:** 1 Department of Pediatrics, The Ohio State University College of Medicine, 700 Children’s Drive, RM FB3322, Columbus, OH 43205 USA; 2 College of Public Health, The Ohio State University, 250 Cunz Hall, 1841 Neil Ave, Columbus, OH 43210 USA; 3 Ohio Department of Medicaid, 50 West Town Street, Suite 400, Columbus, OH 43215 USA; 4 Medical Scientist Training Program, The Ohio State University, Columbus, OH USA

**Keywords:** Medicaid, Social determinants, Population health, Equity, Health insurance, Low-income

## Abstract

**Background:**

Growing understanding of the influence of social determinants of health (SDH) on healthcare costs and outcomes for low income populations is leading State Medicaid agencies to consider incorporating SDH into their program design. This paper explores states’ current approaches to SDH.

**Methods:**

A mixed-methods approach combined a web-based survey sent through the Medicaid Medical Director Network (MMDN) listserv and semi-structured interviews conducted at the MMDN Annual Meeting in November 2017.

**Results:**

Seventeen MMDs responded to the survey and 14 participated in an interview. More than half reported current collection of SDH data and all had intentions for future collection. Most commonly reported SDH screening topics were housing instability and food insecurity. In-depth interviews underscored barriers to optimal SDH approaches.

**Conclusion:**

These results demonstrate that Medicaid leaders recognize the importance of SDH in improving health, health equity, and healthcare costs for the Medicaid population but challenges for sustainable implementation remain.

## Background

State Medicaid programs are tasked with designing, implementing, and co-funding public health insurance systems for low income individuals whose costly healthcare needs are often intertwined with high social needs, such as housing instability or food insecurity. These social needs, often referred to as social determinants of health (SDH), have been shown to drive higher utilization, higher cost, greater health disparities, and poorer health outcomes [1] thus many State Medicaid agencies and Medicaid managed care organizations are testing new approaches for SDH screening, referral, and community partnerships that address such needs directly [2]. These entities have also moved toward integrating social factors into value-based payment models and performance accountability systems designed to promote healthcare quality and health equity [3–5].

While each state’s approach is unique, driven by its own health, financial, social, and political landscape, sharing best practices for SDH data collection and use across states has the potential to help each state optimize its approach, maximizing both value and health. The goal of this project was to identify the current and future SDH priorities of State Medicaid agencies and collect specific examples of delivery system innovation.

## Methods

### Theoretical framework

This study is grounded in the World Health Organization’s Social Determinants of Health Framework which posits that the healthcare system mediates the relationship among intermediary determinates of health (material circumstances, behavioral and biological conditions, and psychosocial factors) and outcomes of equity in health and well-being [6]. Our goal is to characterize the current and planned approaches of State Medicaid agencies to effect that mediation. The WHO defines SDH as the conditions in the environments in which people are born, live, learn, work, play, worship, and age that affect a wide range of health, functioning, and quality-of-life outcomes and risks [7]. In practice, commonly considered SDH have included housing instability, food insecurity, limited access to transportation, income insufficiency, and related social factors.

### Study design

We employed a mixed-methods design, combining online surveys and brief face-to-face semi-structured interviews of Medicaid medical directors, to explore how SDH are being considered within State Medicaid Programs. This project was deemed not to constitute Human Subjects Research by Nationwide Children’s Hospital IRB.

### Population

The Medicaid Medical Directors Network (MMDN) began in 2005 with support from the Agency for Healthcare Research and Quality. It was designed to advance the health of Medicaid patients with a focus on the development and use of evidence-based medicine, measurement and improvement of health care quality, and the redesign of health care delivery systems by bringing together clinician leaders from State Medicaid programs. MMDN participation is open to Medicaid medical directors (MMD) and those in similar clinical leadership positions who advise the Medicaid director for one or more components of a Medicaid program. The MMDN contains 42 member-states, including Washington D.C., and the group meets regularly under the sponsorship of AcademyHealth, sharing insights to common problems. The nine states that were not participants in the MMDN at the time of the survey were geographically diverse including four Western states, two Southeastern states, two Mid-Western states, and one Northeastern state. None had expanded Medicaid at the time of the survey.

### Online survey

An online survey was designed to collect information on present state and desired future state for collection and use of SDH data within Medicaid programs using a list of 10 evidence-based SDH topics identified through a review of previously published literature [8, 9]. The following SDH topics were included: 1) housing instability, 2) utility needs, 3) family and social support, 4) education and/or literacy, 5) food insecurity, 6) employment, 7) transportation needs, 8) criminal justice involvement, 9) intimate partner violence, 10) interpersonal safety. The items and survey design were reviewed by MMDN leaders for face and content validity. A study invitation e-mail with a link to the online survey was sent to all 42 member-states of the MMDN listserv on September 29, 2017. Three survey reminder emails were sent, approximately weekly, in the month preceding the face-to-face meeting to maximize the response rate.

### Semi-structured interviews

Medicaid representatives from 21 states attended the MMDN meeting in November 2017. At this meeting all state representatives present were invited to participate in face-to-face semi-structured interviews, regardless of having completed the online survey. An interviewer and recorder followed a semi-structured interview guide developed by the project team to elicit information about what SDH information was being collected, how it was being collected, and what successes and challenges they would like to share. Each interview lasted between 10 and 20 min.

In addition, if the interviewee had completed the online survey, survey responses were reviewed, and time was provided for additional details on responses. In cases where the survey had not been completed, at the end of the interview respondents were asked the survey questions. When permission was provided, interviews were recorded, and notes were taken. Audio recordings were transcribed verbatim for qualitative analysis.

### Analysis

From the online survey, we calculated the number and percentage of member-states currently collecting information on each SDH topic, directly or through partners, and the number and percentage that planned to do so in the future. We summarized the current and future SDH topics and use of collected SDH data. For the interview responses, thematic analysis of transcribed interviews was used to code recurring themes across member-states. Transcripts were independently reviewed by at least two authors who coded the primary content of each statement then grouped statements into themes. Reviewers then met to discuss identified themes and come to consensus on primary and secondary themes. Representative quotes were identified for each primary theme.

## Results

### Online survey

**Fig. 1:**
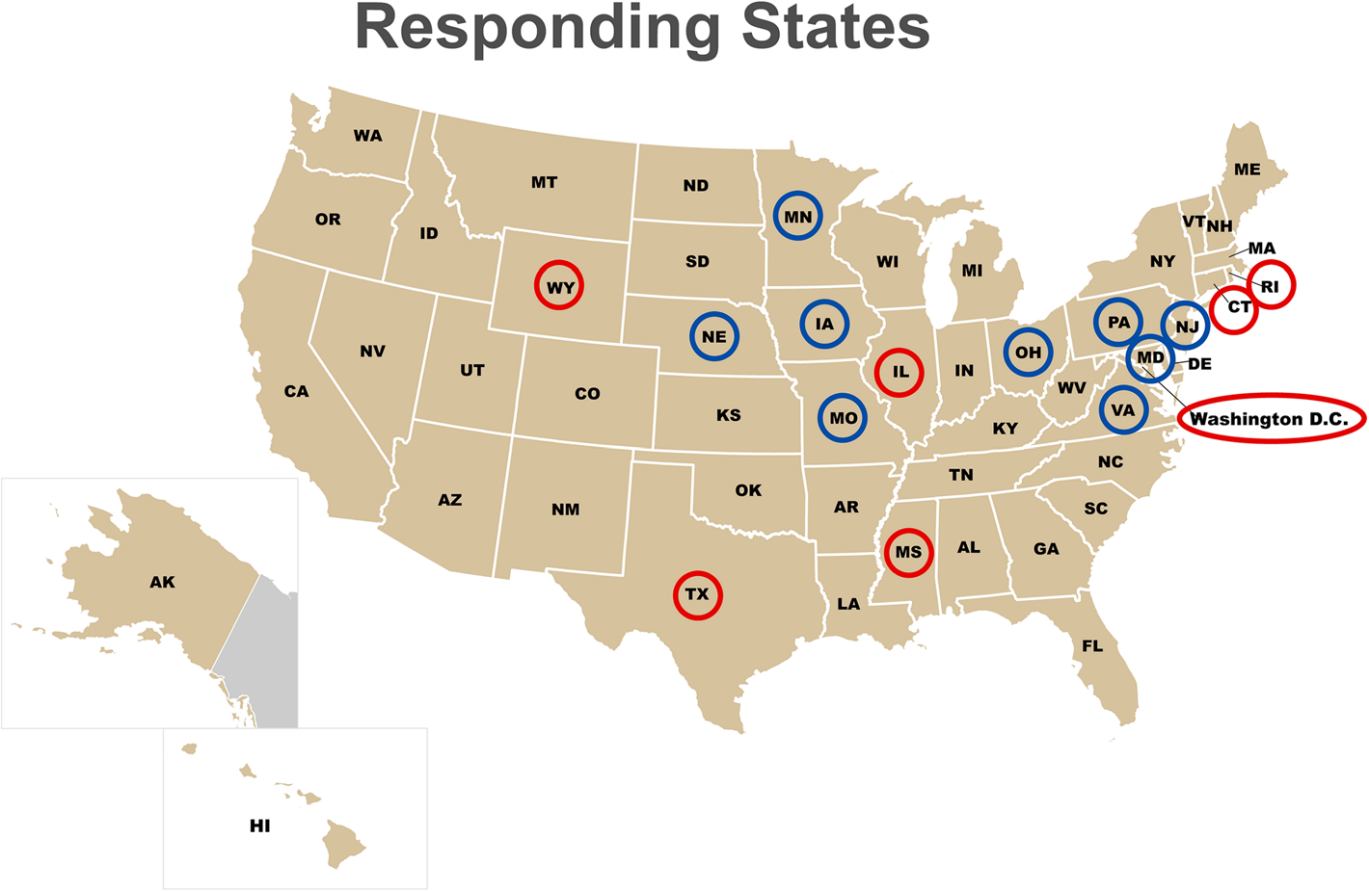
Online survey respondent by current and future SDH-related data collection

Our email survey, distributed to the 42 member-states, generated responses representing 17 states yielding a response rate of 40.5% (Fig. 1). When multiple responses were received from one state, only the response from the medical director was included.

Over half of our respondents (9 member-states) reported that they are currently collecting and using SDH data for their Medicaid programs (Table 1). The most commonly identified topics were housing instability and food insecurity, both over seven member-states (Table 2). All respondents stated the intention to start or continue collection in the future. Topics with the greatest planned growth were criminal justice involvement and intimate partner violence. While some Medicaid programs were directly engaged in SDH data collection many sought data through partnerships with managed care plans, other state agencies, and social service agencies (data not reported).

**Table 1:** Survey respondent characteristics

MMDN participating states	42	
States Included in Analyses	17	40.5
Primary Respondent		
Medical Director	14	82.4
Assistant Medical Director	2	11.8
Other/not reported	1	5.9
Region		
Northeast	4	23.5
Midwest	7	41.2
South	5	29.4
West	1	5.9
Expansion Status		
Expansion	10	58.8
Non-Expansion	7	41.2

**Table 2:** Curent and planned future SDH data collection acivities

SDH Topic	Current	Current or Future
Any	9	17
Housing Instability	9 (100)	15 (88.2)
Food Insecurity	8 (88.9)	15 (88.2)
Transportation	7 (77.8)	14 (82.3)
Education/Literacy	7 (77.8)	13 (76.5)
Criminal Justice Involvement	4 (44.4)	10 (58.8)
Intimate Partner Violence	2 (22.2)	6 (35.3)

Current uses of data varied greatly. Targeting interventions, setting Medicaid population health goals, and setting Medicaid health disparity goals were each endorsed by more than half of member-states (Table 3). Areas with the greatest intended expansion were monitoring managed care plans or providers, incentivizing managed care plan or provider performance, stratifying health outcomes measures, and setting overall population health goals.

**Table 3:** Current and future uses of SDH data

SDH data uses	Current	Current or Future
Any	9	17
Targeting Interventions	6 (66.7)	15 (88.2)
Setting medicaid population health/healthcare disparities goals	6 (66.7)	13 (76.5)
Setting medicaid population health/healthcare goals	5 (55.6)	11 (64.7)
Incentivizing providers/plans	3 (33.3)	11 (64.7)
Monitoring plan performance	3 (33.3)	10 (58.8)
Stratifying health outcomes measures	2 (22.2)	11 (64.7)
Setting overall population health goals	1 (11.1)	7 (41.2)

### Semi-structured interviews

Fourteen of the twenty-one (21) states in attendance at the MMDN meeting participated in semi-structured interviews (67% response rate). The distribution of states across regions was nearly equal: four Northeast member-states, three Midwest member-states, four Southern member-states, and three Western member-states. Seven states completed both the online survey and the semi-structured interviews. One interview was conducted approximately one week after the MMDN meeting in-person due to time constraints at the meeting. Below we discuss three identified themes that provide particular insight to the questions of SDH screening policy and implementation: data, state policy and politics, and financial considerations. Table 4 offers verbatim quotes specific to each of these themes.

**Table 4:** Exemplar quotes for semi-structured interviews

Topic	Exemplar Quote
Data Concerns	*“Um, so we link our vital records data with our Medicaid data for like low birth weight, and for some other race and ethnicity things we it from our vital records because it’s a better source so that, we do that. We have brought in incarceration data. Incarceration data is actually public data so it’s easy to bring in.”*
	*We are not currently collecting. But we already have databases that include social determinants information. We are in the process right now with our new data warehouse of moving data from various systems all into the data warehouse."*
	*“We did a report … where we took all these indicators for children and compiled them into a report and then did a demographic breakdown by race/ethnicity … so we did a multivariate analysis and we have all this data in that database to look at the relationship of child welfare … or child abuse reporting …*”
	“*… in terms of the reimbursement system, so ICD9 and CPT and HCPCs coding, having those appropriate modifiers and having them be applied seamlessly as you’re going through an episode of care in the record where the billing and claims occurs at the point of care and then having that go up to the managed care organizations or to fee for service Medicaid and for that data to come through in a seamless manner without additional burden in terms of QI measures or billing coding procedures that are beyond face-to-face eye-to-eye contact with a beneficiary and further delaying and shrinking the opportunity to have a meaningful conversation. Those are all critical issues that we need to do to align that to occur in the interaction between the electronic medical record, the provider, and the patient; the dialog … And if we can collect that data correctly and record that data correctly, the rest will fall into place, including pay for performance.”*
	*… since they all use all of these different tools we don’t actually have a view across the state, even for that top 2% of how many of them actually have housing issues and food instability, because they all ask a different way, the definition of it might actually be different. So, um, our national quality measures stewards need to get their act together, and actually develop measures that have those kinds of specs like the ones we talked about."*
Policy Landscape	*“You know we have under review right now a 1115 waiver proposal that focuses on community engagement requirements which is a particular interest of the federal administration … a large part of that deals with employment and job availability, so in my mind for a lot of our able bodies expansion population that’s one of the SDH to a large degree, is just if you are going to predicate Medicaid vision and dental benefits and just overall eligibility on fulfilling these community engagement requirements that one way I see a clear connection between a major focus of our state’s Medicaid program – a way we can sustain benefits for an expansion population and then getting outside of what’s been really traditionally conceived as a payable service.”*
	*“But I have to recognize that one of the strategies may be well ‘how do we work with this political engine better?’ And you know I have to confess when I was in practice I just was not connected to really how the engine worked. I was always on the receiving end, you know, just trying to do my best in my little neck of the woods and not really look at how much. And now I’m like oh my goodness we need more engaged clinicians to help be part of a better future for everyone … the SDH solution has to have a political arm to it, otherwise it just dies.”*
Financial Challenges	“*… I think everybody recognizes or verbalizes an understanding of the social determinants of health are a factor. Um, I would like to be able to put a dollar sign on that factor to go to our budget office and beat them over the head with it (laughs).”*
	*“I think the incentive, we’ve talked about that a little bit but I think the incentive I would like to use it for is to incentivize collaboration between health services, medical services, and social services. And then to get people to address that more solidly. Um, and give a return investment to the community itself. That would be the score and that’s something that we’re, I don’t think we have quite the mechanism to do that yet but that would be the right way to do it.”*
	*"And then the other thing is that we need to also encourage that that gain sharing is encouraged down to the level of the provider and maybe even to the patient. Maybe for doing a good job they get a subway card or a card to- a netflix card. Or whatever. Or a movie card. So we currently have that in our Medicaid expansion program. Those who are successful in doing preventative measures or managing chronic conditions, we provide a reward card to minimize their copays and deductibles.*

### Data

Among member-states that are currently collecting SDH, there are significant concerns regarding data quality and data sharing. Leveraging data collected by state agencies other than Medicaid was frequently discussed as a means of minimizing data collection burden and maximizing quality. However, aging data systems and organizational silos were noted as limiting factors. While a few member-states successfully linked data systems across state agencies and managed care plans to create reports, dashboards, and statistical models to assess the extent of SDH and their impacts on health outcomes, this was still an aspirational goal for most member-states seeking to integrate SDH in the next few years.

One noted barrier to interagency sharing and use of SDH data was the lack of standardization of data formats and definitions. Race and ethnicity data was often noted as a problem because different systems use different ascertainment methods (e.g., self-report, case worker report) and different options (e.g., options to select more than one race or ethnicity, options to not report). Incompleteness and inconsistency of race/ethnicity was cited as a barrier to quantifying health disparities and targeting health equity interventions. Concerns were also noted regarding the lack of standardized SDH screening questions leading to different data being collected in different agencies and settings and limiting the quality of evidence available to support the use of SDH data in performance management or incentives.

### Policy and politics

Member-states described both barriers and opportunities associated with the current volatility in the healthcare policy landscape. For example, the opportunity for adding community engagement and work requirements to Medicaid eligibility criteria through the 1115 Waiver program was described as an opening to consider the role of SDH in employability. SDH are a key part of many waiver programs under review or in practice by the member-states interviewed.

On the other hand, it was noted that instability and uncertainty in the health policy environment, makes mid-range and long-term planning difficult and that ideological opposition to Medicaid in some state legislatures and executive branches makes discussion of expanded SDH considerations a “non-starter”. One MMD recalled that as a physician, the politics of Medicaid weren’t a focus but now, being inside the system, she recognized that political engagement from stakeholders is imperative to the design of a program that meets the needs of providers and patients.

### Financial considerations

Tight state budgets were often cited as a barrier to expanded or innovative collection and use of SDH data. It was simply stated that all data collection costs money. There was some discussion of paying for SDH screening at the clinical level (similar to pediatric developmental screening) to maximize data collection but lack of evidence based standardized tools was cited as a barrier. It was noted that higher resourced plans and providers are capturing information now as “good citizens” but smaller and rural providers can’t always take on the expense without payment. One medical director argued that if researchers could determine the real costs associated with NOT screening for and addressing SDH, it would be easier to find and justify the resources to do it. Some member-states are utilizing census tract data enhanced by GIS mapping and other community-level data to develop deeper insights that support the business case for SDH through partnerships with academic institutions. However, even with partnerships cost was repeatedly noted as a challenge.

## Discussion

Many State Medicaid programs use SDH data currently or plan to in the near future as a strategy to address the social drivers of health care expenditures, but barriers to sustainability abound. We found that housing instability and food insecurity were the most often cited areas of data collection and referral. This is not surprising, given the degree of poverty required for Medicaid eligibility and the growing evidence base linking these social needs with healthcare costs and outcomes. These are also areas in which Medicaid agencies and healthcare providers reported having the strongest existing community partnerships. Criminal justice involvement and intimate partner violence were among the least measured SDH but were recognized as important untapped populations and exposures for future assessment. Several MMD reported the desire to expand screening into these areas but privacy, safety, and stigma issue create challenges for both screening and referral.

Current uses of SDH data are descriptive and strategic, specifically targeting interventions and setting population health goals. Our follow-up interviews offered insights into the barriers to moving to the desired next steps of using data for monitoring and incentivizing performance. Primary barriers included cost and quality of data collection, insufficient evidence base for creating performance expectations, and competing priorities These concerns align with insights from executives of Medicaid managed care organizations (MMCO) who are beginning to make investments in SDH interventions but are finding difficulty in clinical integration, financing, evaluation, and sustainability [4].

The content of our interviews yielded four urgent calls to action for advancing approaches to SDH.

1. Development of validated measures of SDH related risks and outcomes – The lack of validated measures limits comparability across plans, providers, and states. MMDs described managed care plans as hesitant to include contract language on SDH-related performance measures without a strong evidence base which begins with a standard definition and voiced a desire for a brief, modular tool developed with broad, multidisciplinary input and proven to be associated with meaningful health outcomes.
2. Reconsideration of privacy and confidentiality policies that block individual level data sharing within and across agencies – Multiple member-states noted that a true patient-centered approach to population health and well-being requires a systems approach that crosses sectors including public and private insurers as well as across behavioral and physical health systems and nontraditional sites of service such as schools, corrections and rehabilitation, drug courts, social services, housing authorities and not-for-profit entities. Employment services, social services, criminal justice, and housing assistance are also key partners. Privacy and confidentiality policies should be re-visited with a focus on the balance between privacy concerns and need for comprehensive continuous services across systems. Approaches that present novel applications of existing technologies like blockchain may advance the field of secure data linkages in a manner that satisfies federal, state and local data stewards.
3. Development of pilots or demonstration projects that allow spending across health and social service silos – A consistent message gleaned from the interviews was that “silos” cannot address complex social needs. One MMD explained this challenge well: “*… I don’t know that piecemeal-ing some of this is going to help. If a hospital says they’re going to do screening for food insecurity and then make a referral for food insecurity, that’s a good thing. That’s a great step in the right direction, but it doesn’t get into a holistic view a person’s needs”.* State Medicaid agencies must partner with other state agencies and with providers, payers, and social service organizations to develop a person-centered rather than a need-centered approach to SDH. For example, efforts in the state of Ohio have demonstrated the potential reach that collaborations hold. Using a collective impact model, leveraging funding from the Medicaid Technical Assistance and Policy Program (MEDTAPP), Ohio’s Department of Medicaid was able to build a collaborative among academic medicine institutions and state agencies to test a model of shared responsibility to reduce infant mortality through the provision of progesterone to high risk women and the training and the utilization of community health workers [10]. Efforts to coordinate can focus on specific neighborhoods (hotspots), the most pressing problem (community-identified priority), or can be oriented at deploying a dedicated cadre of social determinants-oriented community health workers. The federally-funded Accountable Health Communities model, currently being trialed in 31 communities across the country, leverages Medicare, Medicaid, and community-based organization to test cost effectiveness and sustainability of universal SDH screening [11]. Each of these approaches is being tested across the country, and the lessons learned will inform which combination of strategies will affect the cost of care, quality measures, and health outcomes [12].
4. Need for an infrastructure and funding mechanism allowing states to share best practices and conduct research on what works – The MMDN offers the opportunity to develop new insights and share practical approaches to issues common to Medicaid programs across the country. Understanding the complexity of moving parts required for progress in different states provides a route for dissemination of best practices that may accelerate the achievement of more equitable outcomes. Some small, multi-state collaboratives are partnering to advance novel approaches using non-proprietary data sources to build risk-adjustment models that incorporate SDH through neighborhood stress scores, for example (MA) but expanded cross state share could speed innovation. A sustainable funding mechanism for such collective work is lacking, particularly as many entities develop proprietary products limiting scalability within Medicaid programs.

The most notable limitation of this work is the fact that our survey and interview data are representative of fewer than half of State and territorial Medicaid programs. States that do not participate in the MMDN or who did not to respond when invited to participate in the survey and interview may differ from participants. As such, although our group covers over a quarter of programs and includes all four regions of the country, this is not a fully representative sample. The states that we engaged were also diverse in their level of urbanization, their political landscape, and their size. Because this is a self-selected sample, we cannot state that it is “nationally representative” but we are comfortable stating that our findings reflect the diversity of approaches underway. Instead it can be viewed as snapshot of the activities and thoughts of a subset of MMDs with interest in the topic. Because every state’s Medicaid program is unique, generalizability in approach will likely never be the goal. These results can, however, help to inform the overarching policies and structures necessary for each program to be successful in their own context.

## Conclusion

State Medicaid agencies cover healthcare costs for many of the low-income Americans and subsequently can play a role in determining how payers, clinicians and systems address social determinants of health. These agencies can also have a role to play in achieving health equity through payment and delivery system a reform that incorporates SDH considerations. This project identified the SDH priorities of State Medicaid agencies. It also provided new insights regarding factors influencing states’ ability to reach their SDH goals. Barriers identified by the participating MMD should be the next targets of policy designed to improve population health.
